# Comment on Parker *et al*. (*Evolution, Medicine and Public Health* 2021;9:120–30.)

**DOI:** 10.1093/emph/eoab011

**Published:** 2021-04-16

**Authors:** Graham A.W. Rook

**Affiliations:** Centre for Clinical Microbiology, UCL (University College London), London, UK

It is accepted that we are in a state of evolved dependence on exposure to micro- and perhaps macro-organisms. They populate the microbiota and provide metabolites, such as short-chain fatty acids that drive crucial physiological functions. They also provide data to select relevant lymphocyte clones and molecular signals that drive development of the innate and adaptive immune systems together with their crucial immunoregulatory control mechanisms. When these inputs fail the resulting faulty immunoregulation is at least partly responsible for the increased chronic inflammatory disorders that emerge as societies adopt modern western lifestyles. So which inputs are essential for driving immunoregulation? 

Parker *et al.* emphasize [1] helminth infections in this context (plus some mention of protists). They suggest that the absence of helminths in wealthy urbanized societies predisposes not only to dysregulated immune responses and autoimmune disorders but also to increased lethality of COVID-19. There are problems with these assertions.

Evolution often converts the inevitable into a necessity. For example, most mammals can synthesize vitamin C, but in humans and some other species, a crucial enzyme has become corrupted because the diet of evolving humans ‘inevitably’ contained adequate supplies of the vitamin. As a consequence, vitamin C in the diet is now a necessity. But evolution does not turn *intermittent or temporary* exposures into necessities because this leads to gene-environment mismatch when the exposure is absent. Helminth infection, in terms of human evolution, must be regarded as intermittent or temporary. Helminths downregulate inflammation to avoid fatal immunopathology, and Parker *et al*. imply that we are in a state of evolved dependence on this helminth-mediated immunoregulation. I have supported this view in the past, but now I am doubtful. Different helminth species live in blood, tissues, bladder or gut, and each species downregulates inflammatory responses via a different mechanism. Moreover, the loads of helminths differ wildly between individuals, even when they live in similar geographical locations. There is no constant ‘inevitable’ helminth-associated factor that could drive hard-wired genetic dependence on helminths [2]. Rather than becoming written into an invariant dependence, intermittent or temporary environmental or infectious stresses are coped with via epigenetic adaptations in the developing immune system that can fade over several generations, or be renewed if required.

This argument helps us to understand the conflicting results of trials of helminth therapy in multiple sclerosis (MS). Allowing Argentinian MS patients to become infected with helminths they would have encountered in childhood can stop progression of the disease because their immune systems developed in the presence of the same helminths and require their continuing presence for normal function [3]. In sharp contrast, trials of helminth therapy for MS or other autoimmune disorders are failing in locations where helminths have not been endemic for several generations [4]. Evolution does indeed turn the inevitable into a necessity, but it turns the intermittent or temporary into an option via epigenetic adjustments, so the need for helminths in Europe and the USA has faded.

So, it is theoretically unlikely that we are in a state of evolved dependence on helminths. Moreover, it seems unwise to discuss helminths without any reference to the mass of recent work that has identified crucial sources of microbial inputs needed to set up our immune systems. First, it is clear that the microbiota of our mothers (and other family members) is crucial, and lifestyle factors, such as caesarean deliveries, antibiotics and lack of breast feeding that interrupt mother-to-infant transfer correlate with chronic inflammatory and metabolic problems later in life. Secondly, the microbiota of the natural environment also provides signals and spore-forming organisms that drive biomarkers of immunoregulation. The evidence for the importance of exposure to these two sources of microbiota is overwhelming, and disruption of these exposures in wealthy urban societies is exacerbated by bad diet (processed, unvaried, fat, sugar and artificial sweeteners) and by stress and air pollution, all of which modulate the microbiota. These factors seem to explain the increase in chronic inflammatory disorders.

These points are also relevant to the proposition that COVID-19 is more lethal in helminth-deprived countries and is correlated with the prevalence of autoimmunity. This assertion is premature. The initially high case fatality rates (CFR) in European countries, such as the UK, were at least partly due to age, obesity, diabetes and infection spreading into care homes. The crucial fact is that Norway and Finland have particularly high rates of autoimmune disease, but very low COVID-19 CFR of ∼1%.

Parker *et al*. also emphasize the therapeutic efficacy of helminths in animal models of autoimmunity. It is true that helminths can attenuate Experimental Autoimmune Encephalomyelitis (EAE), or the SLE-like condition in (NZB × NZW) F1 hybrid or MRL/lpr mice, but similar effects are induced by essentially anything that modifies the gut microbiota, such as administering Lactobacilli or BCG or a changed diet [5]. The animal work does not suggest that helminths are uniquely relevant. Indeed, as a thought experiment, it would be possible to argue that rather than helminths, it is latent tuberculosis in developing countries (supplemented in some environments by BCG), that reduces autoimmunity and increases resistance to COVID-19, particularly when a recent formal clinical trial found that BCG protects from viral respiratory infections. But here again, as for helminths, there is insufficient evidence.

In summary, I suggest that the immunomodulatory effects of helminths are relevant only to individuals whose immune systems matured in their presence and that these effects are mediated via epigenetic mechanisms that are reversed in future generations if the helminths are absent. The immunoregulatory molecules produced by helminths are interesting and we may find that they have therapeutic value in patients with certain genetic backgrounds suffering from specific chronic inflammatory conditions. There is anecdotal evidence for this from people who have self-treated with helminths. This is how we will exploit helminths in the future. I do not think there is a case for trying to reconstitute the human biome with a helminth component. The theoretical basis is doubtful, we would not know which helminth to use or who would benefit, and the regulatory hurdles would be overwhelming.

**Conflict of interest:** No funding was received for the work involved in writing this correspondence. 

The author has no conflicts of interest.
